# Infection of Domestic Dogs in Peru by Zoonotic *Bartonella* Species: A Cross-Sectional Prevalence Study of 219 Asymptomatic Dogs

**DOI:** 10.1371/journal.pntd.0002393

**Published:** 2013-09-05

**Authors:** Pedro Paulo V. P. Diniz, Bridget A. Morton, Maryam Tngrian, Malika Kachani, Eduardo A. Barrón, Cesar M. Gavidia, Robert H. Gilman, Noelia P. Angulo, Elliott C. Brenner, Richard Lerner, Bruno B. Chomel

**Affiliations:** 1 College of Veterinary Medicine, Western University of Health Sciences, Pomona, California, United States of America; 2 Laboratorio de Medicina Veterinaria Preventiva, Facultad de Medicina Veterinaria, Universidad Nacional Mayor de San Marcos, Lima, Peru; 3 Department of International Health, Johns Hopkins University Bloomberg School of Public Health, Baltimore, Maryland, United States of America; 4 Laboratorio de Investigación de Enfermedades Infecciosas, Departamento de Microbiología, Universidad Peruana Cayetano Heredia, Lima, Peru; 5 Asociación Benéfica Proyectos en Informática, Salud, Medicina y Agricultura, Lima, Peru; 6 School of Veterinary Medicine, University of California, Davis, Davis, California, United States of America; Universidad Peruana Cayetano Heredia, Peru

## Abstract

*Bartonella* species are emerging infectious organisms transmitted by arthropods capable of causing long-lasting infection in mammalian hosts. Among over 30 species described from four continents to date, 15 are known to infect humans, with eight of these capable of infecting dogs as well. *B. bacilliformis* is the only species described infecting humans in Peru; however, several other *Bartonella* species were detected in small mammals, bats, ticks, and fleas in that country. The objective of this study was to determine the serological and/or molecular prevalence of *Bartonella* species in asymptomatic dogs in Peru in order to indirectly evaluate the potential for human exposure to zoonotic *Bartonella* species. A convenient sample of 219 healthy dogs was obtained from five cities and three villages in Peru. EDTA-blood samples were collected from 205 dogs, whereas serum samples were available from 108 dogs. The EDTA-blood samples were screened by PCR followed by nucleotide sequencing for species identification. Antibodies against *B. vinsonii berkhoffii* and *B. rochalimae* were detected by IFA (cut-off of 1∶64). *Bartonella* DNA was detected in 21 of the 205 dogs (10%). Fifteen dogs were infected with *B. rochalimae*, while six dogs were infected with *B. v. berkhoffii* genotype III. Seropositivity for *B. rochalimae* was detected in 67 dogs (62%), and for *B. v. berkhoffii* in 43 (40%) of the 108 dogs. Reciprocal titers ≥1∶256 for *B. rochalimae* were detected in 19% of dogs, and for *B. v. berkhoffii* in 6.5% of dogs. This study identifies for the first time a population of dogs exposed to or infected with zoonotic *Bartonella* species, suggesting that domestic dogs may be the natural reservoir of these zoonotic organisms. Since dogs are epidemiological sentinels, Peruvian humans may be exposed to infections with *B. rochalimae* or *B. v. berkhoffii*.

## Author Summary

*Bartonella* are bacteria transmitted by fleas, ticks, sandflies and other insects capable of infecting humans, domestic animals, livestock and wildlife, including marine mammals. In humans, they cause diseases such as trench fever, cat scratch disease, endocarditis, fever of unknown origin and have been recently associated with neurologic and neurocognitive abnormalities. *Bartonella bacilliformis* was first described in Peru in 1913, and it has never been detected in animals. Despite the fact that 14 other *Bartonella* species have been detected infecting humans around the world, no other *Bartonella* species has yet been described from Peruvian humans or domestic animals. We documented a significant number of healthy domestic dogs in Peru infected or exposed to two *Bartonella* species (*B. rochalimae* and *B. vinsonii* subsp. *berkhoffii*), which are known to cause disease in humans. These same species were previously detected in human fleas and dog ticks in Peru, suggesting that vector transmission between dogs and humans may be possible. While the role of dogs as a source of *Bartonella* species for direct transmission to humans is not well understood, preventive measures including vector control in dogs should be implemented to prevent human infection.

## Introduction

*Bartonella* species are gram-negative bacteria associated with an increasing array of disease manifestations in humans and animals. They are small, obligate intracellular organisms that adhere and invade erythrocytes and endothelial cells of mammalian hosts, causing long lasting bacteremia [1], [2]. These zoonotic organisms are mainly transmitted by blood-sucking arthropod vectors, including fleas, body lice, ticks, sandflies and others [1]. To date, 15 species of *Bartonella* are known to infect humans. Among these, nine species have been documented in dogs, based on culture isolation or DNA-based methods: *B. clarridgeiae*, *B. elizabethae*, *B. henselae*, *B. koehlerae*, *B. quintana*, *B. rochalimae*, *B. vinsonii* subsp. *berkhoffii* (hereafter *B. v. berkhoffii*), *B. volans* (including *volans-like*) and *B. washoensis* [3], [4].

*B. bacilliformis* is the most frequent species of *Bartonella* in Peru. Humans are considered the reservoir host and infection in animals has not been reported [1]. No other species of *Bartonella* have been detected from Peruvian humans to date. However, a new bacteria, *B. rochalimae*, was isolated in 2007 from an American woman who became sick 16 days after returning from a 3-week trip to Peru, where she received numerous insect bites [5]. Since this first report, *B. rochalimae* has been detected by culture and/or molecular techniques from three asymptomatic rural dogs in California [6], one stray dog in Colombia [7], one sick dog in Greece [8] and one dog with endocarditis in California [9]. In addition, an experimental infection of dogs, cats, and guinea pigs with *B. rochalimae* demonstrated that only dogs became highly bacteremic without any disease expression, suggesting that dogs could be the natural reservoir for this species [10].

Domestic dogs may represent excellent epidemiological sentinels for *Bartonella* infection in humans due to several factors: exposure to similar household and recreational environments of humans, potential parasitism by the same vectors, wide diversity of *Bartonella* species identified in canines, development of a strong organism-specific antibody response to many vector-borne pathogens; and accessibility for safe handling and sample collection [11], [12]. Therefore, this study aimed to determine the potential for human exposure to zoonotic *Bartonella* species by defining the serological and molecular prevalence of these pathogens in asymptomatic domestic dogs in various geographic regions of Peru. Additionally, this study sought to define the genetic relationship among *Bartonella* species, subspecies and strains detected from Peruvian dogs and previously described *Bartonella* species from humans and other hosts from Peru and other countries. We have demonstrated that Peruvian dogs are exposed to zoonotic *Bartonella* species, and this study may suggest that the human population is at risk of infection with the same species detected by DNA amplification and genetic characterization.

## Materials and Methods

### Ethical statement

All animals were humanely treated during sample collection. Dogs were manually restrained during blood withdraw, in accordance with the rules of the Medical Ethics and Animal Care Committee of the Universidad Nacional Mayor de San Marcos (UNMSM), Lima, Peru (no protocol number was assigned), which adheres to the Public Health Service (PHS) policies of the Office of Laboratory Animal Welfare, Office of Extramural Research, National Institutes of Health (OLAW-OER-NIH), USA (PHS approved assurance number A5934-01). In addition, this study was also approved by the Medical Ethics and Animal Care Committee of the Universidad Peruana Cayetano Heredia (UPCH), Lima, Peru (study protocol number 60501), in accordance with the guidelines of the PHS policies, OLAW-OER-NIH, USA (PHS number A5146-01). The purpose of the study was explained to each individual and they were informed that participation was voluntary and data collected were confidential. Informed consent was obtained from all owners of enrolled dogs in the oral format, because some participants were illiterate. In a pilot study of blood cultures of five dogs where *Bartonella* DNA was detected (data not shown) either lack of isolation of *Bartonella* or major bacterial contamination was observed. Therefore, the present study only tested canine Peruvian blood samples by DNA-based methods.

### Study sites

**Figure 1:**
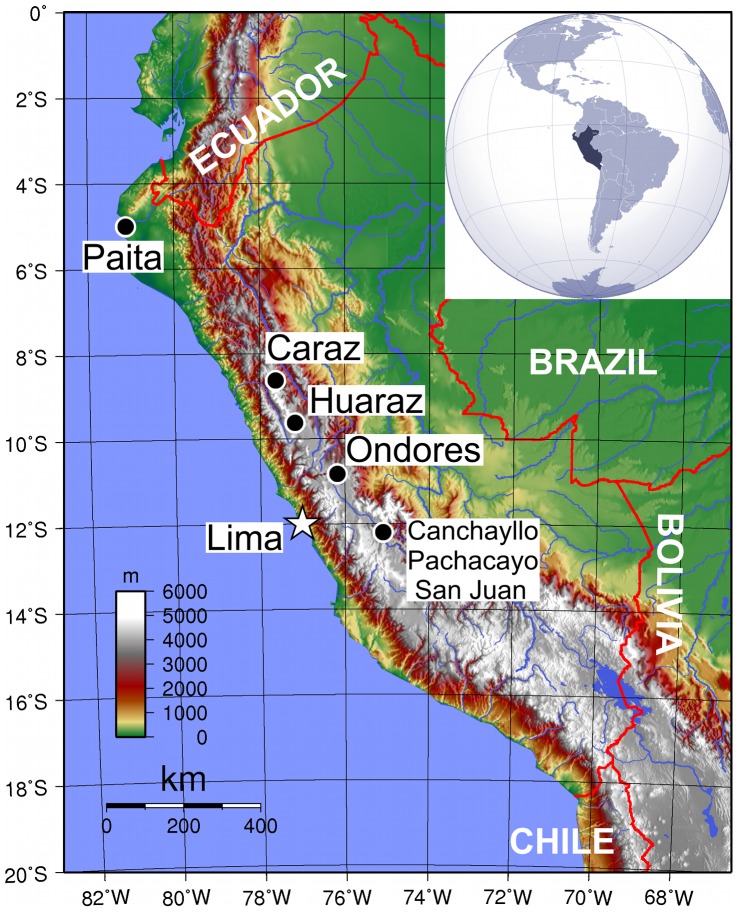
Map of Peru with the location of each study site. The country's capital (Lima) is indicated with a star. The color scale indicates the altitude in meters. The country's borders are delimited in red. Surrounding countries are labeled in white. The insert shows the location of Peru in South America.

**Figure 2:**
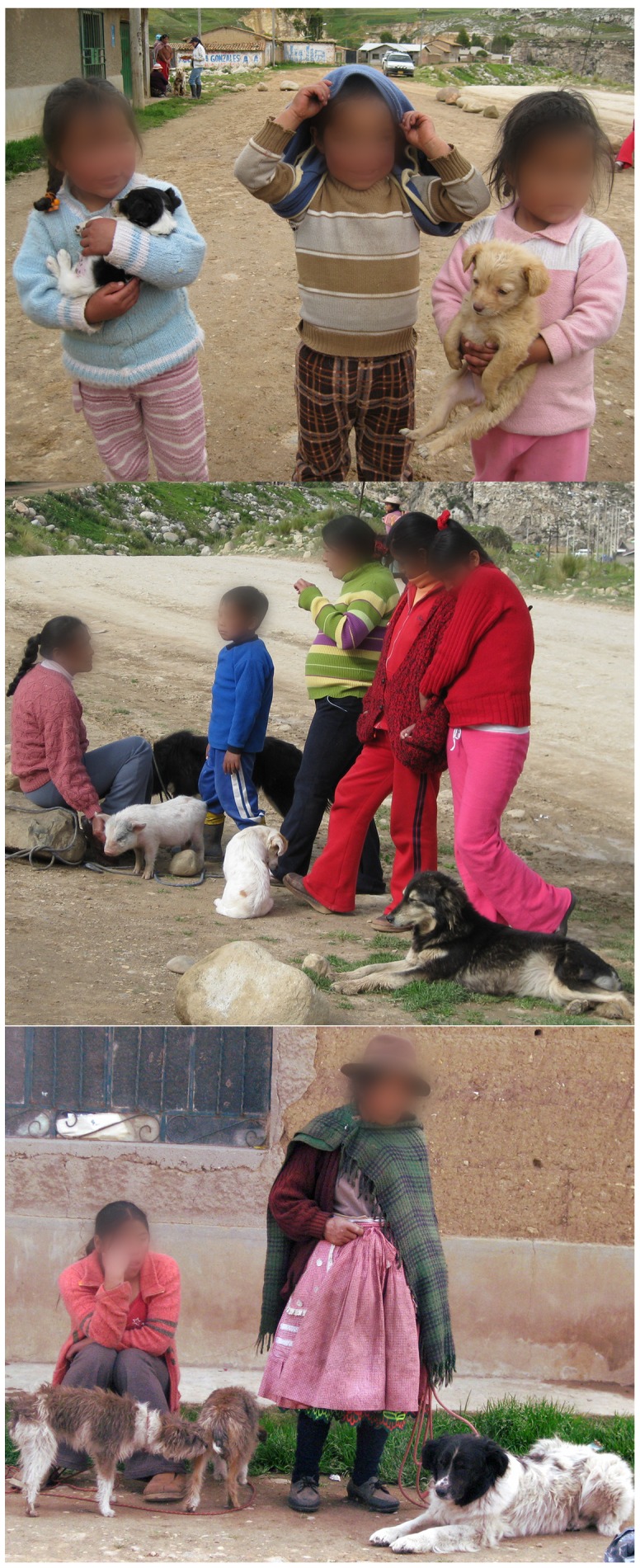
Underserved community at the highlands of Peru. Images illustrate the strong human-animal bond, the number of dogs and other companion animals, and the limited circumstances of the population at one of the study sites in Peru.

This was a cross-sectional molecular and serology survey of a convenient sample of dogs from several geographically-diverse areas of Peru naturally exposed to *Bartonella* species. It was conducted in five cities and three small communities located in four distinct districts of Peru (Figure 1). Locations were selected as part of a study on canine echinococcosis in rural underserved communities in the Central Peruvian highlands (Figure 2), as well as another study on canine ehrlichiosis in underserved communities of Lima (the capital) and in other regions of the country. San Juan de Miraflores (12°9′5″S, 76°58′12″W), a district of the Lima province, is located at the center of the coast at an altitude of 141 m (463 ft.) with a population of approximately 336,000 people. Paita (5°5′6″S, 81°5′58″W), in the Piura region, is located 1,089 km (677 miles) northwest of Lima, at the sea level with a population of approximately 98,000 people. Huaraz (9°31′42″S, 77°31′46″W), in the Ancash region, is located 414 km (257 miles) northeast of Lima, at an altitude of 3,052 m (10,013 ft.) with a population of approximately 53,000 people. Caraz (9°2′54″S, 77°48′54″W), also located in the Ancash region, is located 460 km (285 miles) northeast of Lima, at an altitude of 2,256 m (7,402 ft.) with a population of approximately 20,000 people. Ondores (11°5′11″S, 76°8′46.53″W), in the Junín region, is located 230 km (143 miles) east of Lima at an altitude of 4,105 m (13,541 ft.) with an estimated population of 2,571 people. Canchayllo, Pachacayo, and San Juan de Pachacayo are three small villages (approximately 11°47′56″S, 75°32′37″W), also in the Junín region, located approximately 200 km (124 miles) from Lima at an altitude of 3,671 m (12,043 ft.) with a combined estimated population of 1,774 people [13]. From the four regions where this study was conducted, autochthonous cases of human bartonellosis caused by *B. bacilliformis* were frequently reported, representing over 80% of the all notified human cases in that country between 1950 and 2000 [14].

### Study population and data availability

A convenient sample of 219 domestic dogs was included in this study. In December 2009, 122 dogs from Ondores, Canchayllo, Pachacayo, and San Juan de Pachacayo were enrolled in this study, whereas in December 2010, 97 dogs from San Juan de Miraflores, Paita, Huaraz, and Caraz were enrolled. Healthy dogs were volunteered by their owners for the study. Aggressive dogs, small puppies, and dogs exhibiting clinical signs of disease were not included in this study. The number of dogs per city and village is provided in Table 1. EDTA blood and/or whole-blood samples to obtain serum were aseptically collected from the jugular or cephalic veins, aliquoted, and stored at −20°C until analysis. EDTA blood and serum samples were not available from all dogs enrolled in this study (Table 1). Concomitant EDTA blood and serum samples were available from 94 dogs. Age information was available from 178 dogs, whereas gender information was documented from 202 dogs. The average age was 2.5 years (SD: 2.75 years, 95% CI: 2.1–2.9 years, median: 1.5 years, range: 2 months–14 years). Males represented 58.4% (118/202) of the dogs, whereas females represented 41.6% (84/202). Samples were shipped frozen on dry ice to the College of Veterinary Medicine at Western University of Health Sciences, Pomona, CA, USA (WesternU), under the import permit number 2009-12-105 from the Center for Diseases Control and Prevention, USA.

**Table 1:** Number of blood and serum samples obtained from dogs from each city/village.

	EDTA-blood	Serum	Enrolled Dogs
Study site	N	N	N
Canchayllo	33	34	39
Caraz – Huaraz	39	N/A	39
Ondores	28	28	28
Pachacayo – San Juan	47	46	55
Paita	28	N/A	28
San Juan de Miraflores (Lima)	30	N/A	30
Total	205	108	219

N/A: samples not available from this site.

### DNA extraction, PCR amplification, and DNA sequencing

DNA of samples from Ondores, Canchayllo, Pachacayo, and San Juan were purified using a column-based method (Quick-gDNA Blood MiniPrep, Zymo Research, Irvine, CA, USA) at WesternU. DNA of samples from San Juan de Miraflores, Paita, Huaraz, and Caraz were purified at UPCH using a phenol-chloroform method.

A conventional PCR assay designed to amplify a fragment (approximately 700 bp) of the 16S–23S ribosomal RNA (rRNA) intergenic transcribed spacer (ITS) of *Bartonella* species was performed as previously described [15]. The negative control consisted of molecular-grade water. In order to prevent contamination, sample extraction, reaction setup, PCR amplification, and amplicon detection were performed in separate areas. Quantified genome equivalents (GE) of the DNA from *B. quintana* strain ND1 (DQ648598) [16] were serially diluted 10-fold from 1,000,000 to 1 GE/uL and used as positive control to determine the limit of detection of the PCR assay. This conventional ITS PCR assay was able to detect 50, 25, 10 and 5 GE of *B. quintana* per reaction tube 100% of the time (10/10 times). Canine samples with DNA amplification at the expected size were further genetically characterized by the amplification and DNA sequencing of a fragment (approximately 610 bp) of the heat-shock protein gene (groEL) as previously described [17].

Amplicons were purified from PCR products or from specific bands on the gel (MiniElute kit, Qiagen, Valencia, CA, USA) and sequenced with a fluorescence-based automated sequencing system (Eurofins MWG Operon, Huntsville, AL, USA). Chromatogram evaluation, primer deletion and sequence alignment were performed (Vector NTI Suite 10.1, Invitrogen Corp., Carlsbad, CA, USA). Bacteria species and strain were defined by comparing similarities with other sequences deposited in the GenBank database using BLAST [18]. Phylogenetic analysis of the data was carried out by using the Maximum Likelihood method based on the Kimura 2-parameter model [19] with the MEGA5 software [20]. Bootstrap replicates were performed to estimate the node reliability of the phylogenetic trees, with values obtained from 1,000 randomly selected samples of the aligned sequence data. DNA amplification and sequencing analysis were performed at the WesternU.

### Serological analysis

Antibodies against *Bartonella vinsonii* subsp. *berkhoffii* genotype I (ATCC 51672 [21]) and *B. rochalimae* (isolate Hoopa Fox8, University of California, Davis, CA, USA [9]) were detected using an indirect immunofluorescent antibody assay (IFA), as previously described [6]. All IFA slides were prepared the same way by infecting Vero cells with one of the strains listed above. Samples with detectable fluorescence at a dilution of 1∶64 were considered positive, with endpoint titers being determined thereafter. Negative and positive control samples were included on each slide. Serology assays were performed at the Veterinary Public Health Laboratory, School of Veterinary Medicine, University of California, Davis, CA, USA.

### Statistical analysis

Molecular prevalence of *Bartonella* species and seroprevalence against *B. v. berkhoffii* and *B. rochalimae* are described as absolute frequencies, percentages and 95% confidence intervals (computed using score method). Contingency table analyses were performed to evaluate association with age or gender and any difference in prevalence by geographical region by using the Fisher's exact test for 2×2 comparisons and the Fisher-Freeman-Halton test for row×column comparisons. Contingency table analysis was also performed to evaluate the correlation between serology and PCR results by Cohen's Kappa agreement test. A p-value<0.05 was considered statistically significant. Data analysis was performed using JMP Pro 10 (SAS Institute Inc., Cary, NC, USA).

## Results

**Figure 3:**
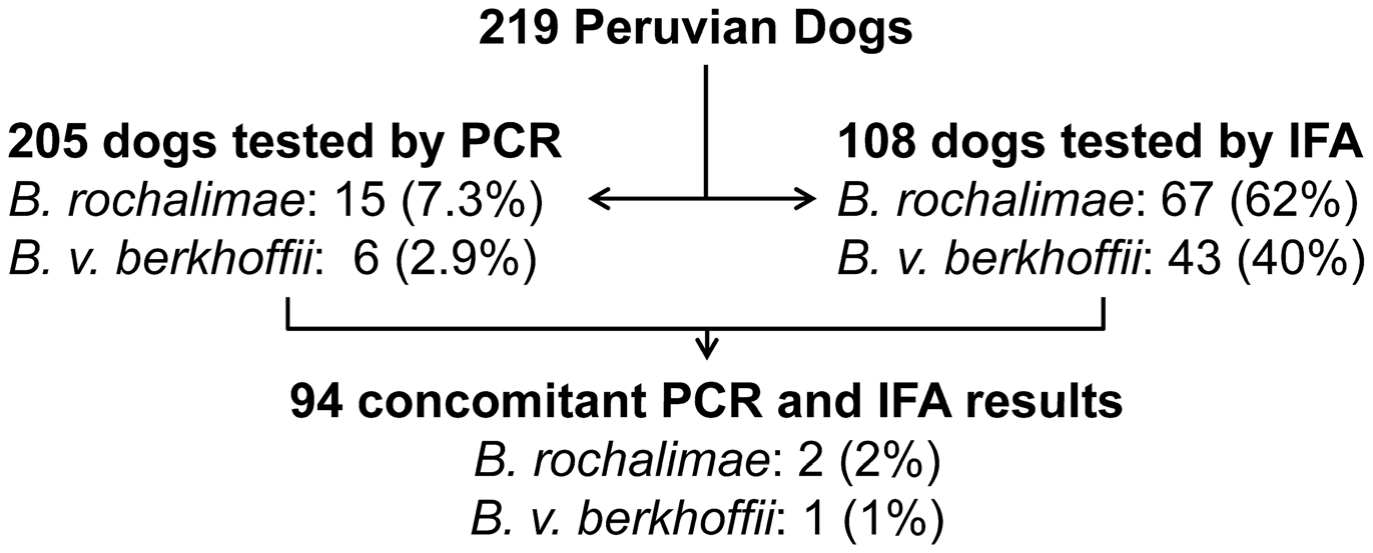
Flowchart of molecular and serological results from Peruvian dogs. *Bartonella* DNA was detected by polymerase chain reaction (PCR), whereas anti-*Bartonella* antibodies were detected by indirect immunofluorescent antibody assay (IFA). Percentages represent proportion of bacteremic and/or seropositive dogs in each sub-group.

Based on DNA amplification and sequence of the ITS region, 15 out of 205 dogs (7.3%) were infected with *B. rochalimae* and 6 of 205 (2.9%) were infected with *B. v. berkhoffii* (Figure 3). Co-infection with both species was not detected in any subject, and no other species of *Bartonella* was identified in this canine population. No significant differences in frequency of *B. rochalimae* or *B. v. berkhoffii* were detected among study sites (respectively p = 0.064 and p = 0.363, Fisher-Freeman-Halton test, Table 2). In addition the frequency of dogs infected with *B. rochalimae* or *B. v. berkhoffii* did not vary between the coastal cities and the highlands of Peru (respectively p = 0.371 and p = 0.187, Fisher's Exact test).

**Table 2:** Frequency of dogs bacteremic^a^ for *Bartonella* species in selected cities or villages in Peru.

	*B. rochalimae*		*B. vinsonii* subsp. *berkhoffii*	
Study site	N/total tested (%)	95% CI	N/total tested (%)	95% CI
Canchayllo	0/33 (0)	0–7.6	0/33 (0)	0–7.6
Caraz – Huaraz	4/39 (10.3)	2.9–24.2	3/39 (7.7)	2.7–20.3
Ondores	2/28 (7)	2.0–22.7	1/28 (3.6)	0.6–17.7
Pachacayo – San Juan	3/47 (6.4)	2.2–17.2	2/47 (2.1)	0.4–11.1
Paita	1/28 (3.6)	0.6–17.7	0/28 (0)	0–8.8
San Juan de Miraflores (Lima)	5/30 (16.7)	7.3–33.7	0/30 (0)	0–8.3
Total	15/205 (7.3)	4.5–11.7	6/205 (2.9)	1.4–6.2

a By polymerase chain reaction assay and DNA sequencing.

CI: confidence interval.

Note: no dog was co-infected with both species of *Bartonella*.

**Figure 4:**
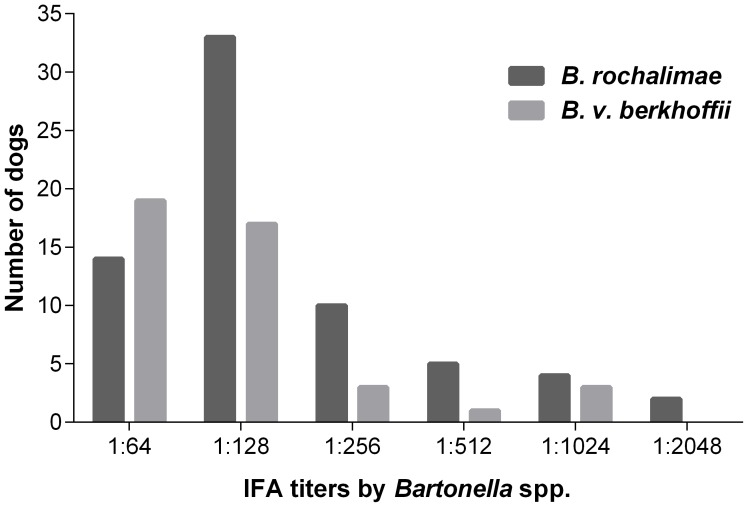
Prevalence of antibodies to *B. rochalimae* and *B. vinsonii* subsp. *berkhoffii* in Peruvian dogs. Antibodies were detected by indirect immunofluorescent antibody assay (IFA). Titers lower than 1∶64 were considered negative and were not included in this figure.

Sixty-seven of 108 dogs (62%) had antibodies against *B. rochalimae* and 43 of the 108 dogs (40%) had antibodies against *B. v. berkhoffii* by IFA (Figure 3) using the standard cut-off of 1∶64. At titer 1∶256 or higher, 21 dogs (19%) were seroreactive for *B. rochalimae* and seven dogs (6.5%) were seroreactive for *B. v. berkhoffii*. Dogs seropositive for *B. v. berkhoffii* were also seropositive for *B. rochalimae*; however, the frequency of dogs seroreactive for *B. rochalimae* was higher than dogs seroreactive for *B. v. berkhoffii* from titer 1∶128 until 1∶2048 (Figure 4). No statistical differences were detected in observed seroprevalence among study sites for *B. rochalimae* or *B. v. berkhoffii* (respectively p = 0.105 and p = 0.773, Fisher-Freeman-Halton test, Table 3).

**Table 3:** Frequency of dogs seroreactive^a^ for *Bartonella* species in selected cities or villages in Peru.

	*B. rochalimae*		*B. vinsonii* subsp. *berkhoffii*	
Study site	N/total tested (%)	95% CI	N/total tested (%)	95% CI
Canchayllo	21/34 (61.8)	45.0–76.1	13/34 (38.2)	23.9–55.0
Ondores	13/28 (46.4)	29.5–64.2	10/28 (35.7)	20.7–54.2
Pachacayo – San Juan	33/46 (71.7)	57.5–82.7	20/46 (43.5)	30.2–57.8
Total	67/108 (62.0)	^52.6^–^70.6^	43/108 (39.8)	31.1–^49.2^

a By indirect immunofluorescent antibody assay.

CI: confidence interval.

Among 108 dogs with IFA results, 43 were seroreactive for both *B. rochalimae* and *B. v. berkhoffii* (Table 4). The highest titer detected for *B. rochalimae* was 1∶2048 (two dogs), whereas for *B. v. berkhoffii* the highest titer detected was 1∶1024 (three dogs). Ninety four dogs had concomitant PCR and IFA results. Among 15 dogs bacteremic for *B. rochalimae* by PCR, only three had serum available for testing, with two dogs seropositive for this species at titers 1∶256 and 1∶512. Conversely, among 6 dogs bacteremic for *B. v. berkhoffii* by PCR, only two had serum available for testing, with one dog being seropositive at titer 1∶128 (Figure 3).

**Table 4:** Number of dogs with positive titers for *B. rochalimae* and/or *B. vinsonii* subsp. *berkhoffii* by indirect immunofluorescent antibody.

	*B. rochalimae* titer (%)							
Bvb titer (%)	Negative^a^	1∶64	1∶128	1∶256	1∶512	1∶1024	1∶2048	Total
Negative	40	11	14					65 (60)
1∶64		2	11	2	2	1	1	19 (18)
1∶128		1	6	7	1	1	1	17 (16)
1∶256			2			1		3 (3)
1∶512					1			1 (1)
1∶1024				1	1	1		3 (3)
Total	40 (37)	14 (13)	33 (31)	10 (9)	5 (5)	4 (4)	2 (2)	108

a Titers lower than 1∶64 where considered negative.

Seropositivity for *B. rochalimae* was associated with adult age (characterized as ≥1 year old) at the time of sample collection, with antibodies against *B. rochalimae* detected in 71% of 69 adult dogs and 46% of 39 young dogs (p = 0.013, Fisher's Exact test). Conversely, bacteremia for *B. rochalimae* was associated with young age (characterized as <1 year old), with *B. rochalimae* DNA detected in 16% of 50 young dogs and 1.8% of 114 adult dogs (p = 0.001, Fisher's Exact test). No association was detected between *B. v. berkhoffii* and age, and no association was detected between sex and serology or PCR results for both *Bartonella* species.

### Phylogenetic analysis

**Figure 5:**
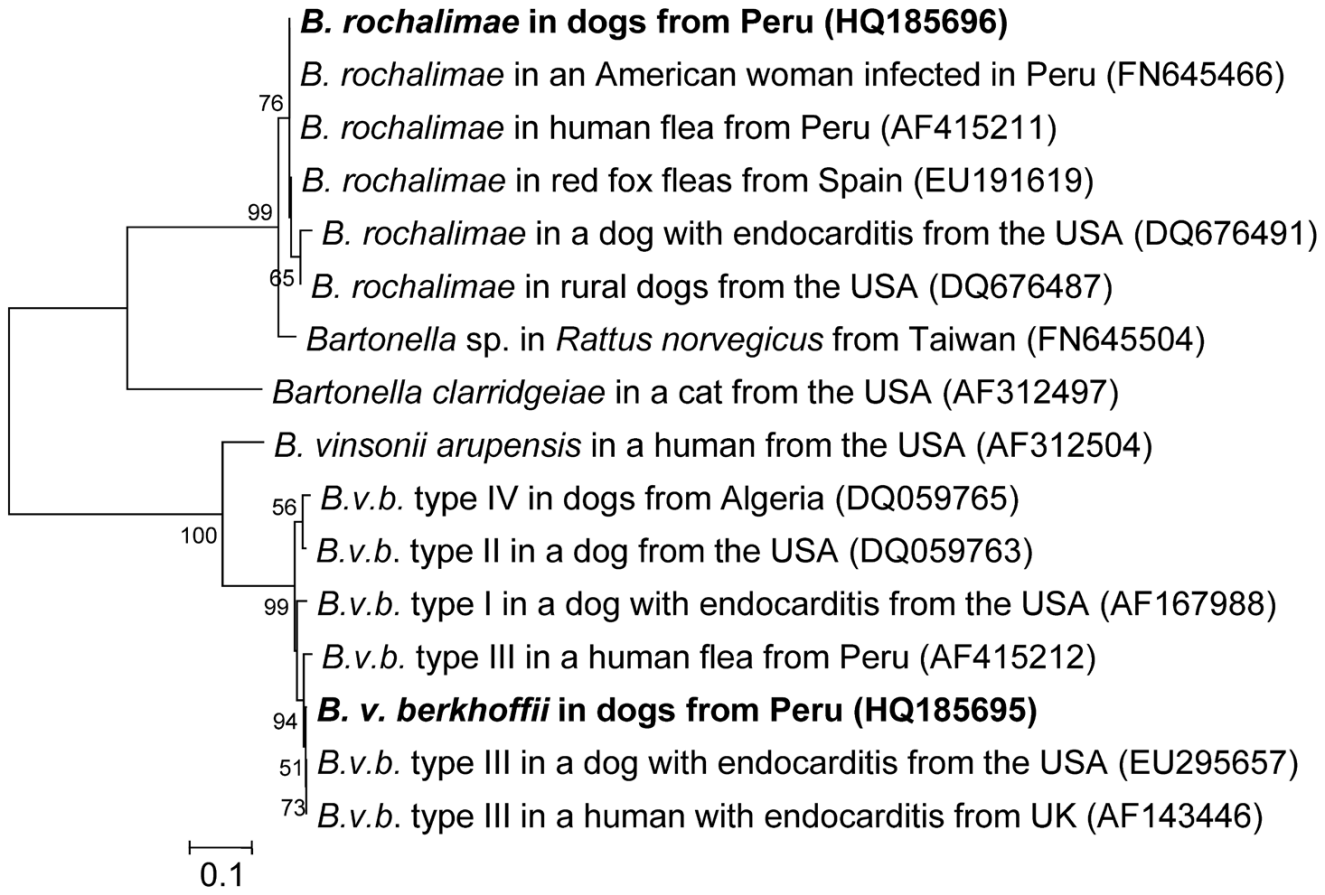
Phylogenetic tree of the 16S–23S rRNA ITS region of *Bartonella* species. The phylogenetic tree was based on 534 bp sequences of the 16S–23S rRNA Intergenic Transcribed Spacer (ITS) of *Bartonella rochalimae* and 621 bp sequences of *Bartonella vinsonii* subsp. *berkhoffii (B.v.b.)* from Peruvian dogs (in **boldface**) and closely related organisms by using the Maximum Likelihood method based on the Kimura 2-parameter model. Each bacterial name is followed by the isolation source and geographic origin, and the GenBank accession number is provided in parentheses. The numbers at the nodes indicate percentages of bootstrap support based on 1,000 replicates. Percentages corresponding to partitions reproduced in fewer than 50% of bootstrap replicates are collapsed. The scale bar indicates 0.1 substitutions per nucleotide position. Primer regions were deleted from sequences. Phylogenetic analyses were conducted in MEGA5 [20].

Multiple DNA sequence alignment and phylogenetic analysis of ITS region from Peruvian dogs (Figure 5) demonstrated that DNA sequences from 15 dogs were 100% homologous (534/534 bp) to the original *B. rochalimae* isolate obtained from the sick woman (ATCC isolate BAA-1498 [5]). This ITS sequence from Peruvian dogs (HQ185696) was also 100% homologous (534/534 bp) to a previously reported DNA sequence from a flea (*Pulex* sp.) collected in 1998 from a person in the city of Cusco, Peru [22], but different (532/534 bp) from DNA sequences from *B. rochalimae* detected from fleas (*Pulex irritans*) from red foxes (*Vulpes vulpes*) in Andalusia, Spain [23] and from dogs and gray foxes (*Urocyon cinereoargenteus*) in northern California (531/534 bp) [9].

A 621 bp sequence of ITS region of *B. v. berkhoffii* was obtained from six Peruvian dogs. Phylogenetic analysis indicated a high homology with DNA sequences from genotype III (Figure 5). Similar to *B. rochalimae* sequences from this study, the DNA sequence of *B. v. berkhoffii* from Peruvian dogs (HQ185695) was 99.6% homologous (562/564 bp) to a previously reported DNA sequence from another flea (*Pulex* sp.) collected in 1998 from another human in Cusco, Peru [22]. *B. v. berkhoffii* sequence from Peruvian dogs was 99.7% homologous (619/621 bp) to *B. v. berkhoffii* genotype III isolate obtained from a human with endocarditis from the United Kingdom [24], and a dog with endocarditis from the USA [25].

**Figure 6:**
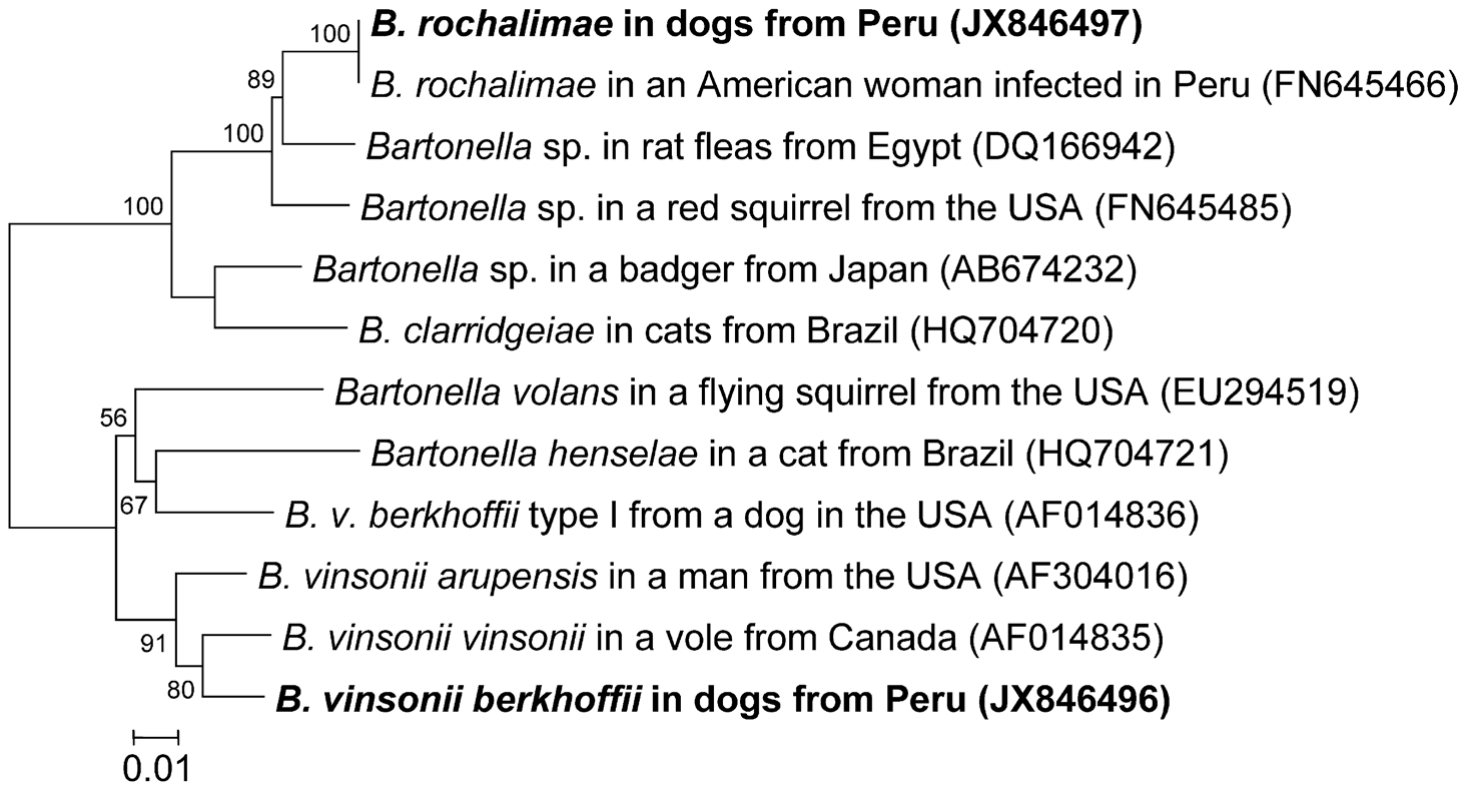
Phylogenetic tree of the 60 kDa groEL gene of *Bartonella* species. The phylogenetic tree was based on based on 565 bp sequences of the 60 kDa heat shock protein gene (groEL) of *Bartonella rochalimae* and *Bartonella vinsonii* subsp. *berkhoffii* from Peruvian dogs (in **boldface**) and closely related organisms by using the Maximum Likelihood method based on the Kimura 2-parameter model. Each bacterial name is followed by the isolation source and geographic origin, and the GenBank accession number is provided in parentheses. The numbers at the nodes indicate percentages of bootstrap support based on 1,000 replicates. Percentages corresponding to partitions reproduced in fewer than 50% of bootstrap replicates are collapsed. The scale bar indicates 0.1 substitutions per nucleotide position. Primer regions were deleted from sequences. Phylogenetic analyses were conducted in MEGA5 [20].

A 614 bp sequence of groEL gene was obtained from eight out of 15 dogs infected with *B. rochalimae* (JX846497) being 100% homologous (565/565 bp) to the original *B. rochalimae* isolate obtained from the sick woman [5] (Figure 6). The groEL sequence from Peruvian dogs was also 96.8% homologous (547/565 bp) to a *Bartonella* sp. sequence detected from rat fleas from Egypt [26] and it was 96.5% homologous (545/565 bp) to a *Bartonella* sp. isolated from an American red squirrel [27]. Other groEL sequences of *B. rochalimae* were not available in GenBank database at the time of writing. A 614 bp sequence of groEL gene was obtained from two out of the six dogs infected with *B. v. berkhoffii* (JX846496) being 97.2% homologous (549/565 bp) to *B. vinsonii* subsp. *vinsonii* isolate obtained from a vole from Canada (strain Baker, ATCC VR-152), and 96.3% homologous (544/565 bp) to *B. arupensis* isolate obtained from an American cattle rancher with bacteremia (ATCC 700727 [28]). However, the canine Peruvian sequence of *B. v. berkhoffii* was only 94.3% homologous to *B. v. berkhoffii* genotype I isolated from a dog with endocarditis in the USA (ATCC 51672 [21]). Further comparison with genotypes II, III, and IV was not possible due to lack of those groEL DNA sequences in GenBank database at the time of writing.

## Discussion

This is the first report of molecular detection of *Bartonella* species in dogs in Peru, documenting the largest number of domestic dogs infected with or exposed to zoonotic *Bartonella* species to date. Our study documented that over half of the canine population tested was exposed to *B. rochalimae* with 1 in every 14 asymptomatic dogs being infected with this zoonotic organism. It is suggested that the natural reservoirs of *B. rochalimae* are gray foxes, coyotes and raccoons in the United States [6], [29], red foxes in Europe [6], [29], [30], and possibly rodents in Asia [31]. Experimentally, dogs are more permissive than cats or guinea pigs to infection with *B. rochalimae* [10]; however, prior to this publication, only a very limited number of naturally-infected dogs were reported [6]–[9]. A significant number of seropositive dogs was detected in this study, with one in five dogs presenting reciprocal titers ≥1∶256, suggesting that these companion animals are frequently exposed to this zoonotic organism, and indirectly suggests that the human population may be at risk of infection as well. DNA sequencing of two distinct regions demonstrated that *B. rochalimae* from Peruvian dogs was 100% similar to the original isolate of *B. rochalimae* from a sick woman, supporting the zoonotic potential of this organism in Peruvian dogs. The majority of dogs enrolled in this study were located in underserved rural communities or in low-income areas of cities (Figure 2), where ectoparasite control and access to veterinary care are very limited. Collective, the current data suggest the life cycle of this zoonotic pathogen is intrinsically related to dogs, vectors, and humans and may be naturally taking place in the study areas. Of relevance is the fact that all seropositive and/or bacteremic dogs for *B. rochalimae* in our study were asymptomatic, which was previously demonstrated in an experimental infection study in dogs [10]. The lack of clinical signs poses an epidemiological challenge since these dogs may serve as reservoirs for extended periods of time without the need to seek veterinary care, which would provide the opportunity for diagnostic investigation of this and other vector-borne zoonotic diseases.

Additionally, approximately 40% of the dogs in this study were exposed to *B. v. berkhoffii*, with 6 dogs infected with this pathogen. In contrast, antibodies to *B. v. berkhoffii* are infrequently detected (<4%) in sick referral or healthy (<1%) dogs in the USA [1]. However, over three quarter of the Peruvian dogs seropositive to this organism had antibody titers between 1∶64 and 1∶128. Possible explanations would include past exposure to *B. v. berkhoffii*, modulation of the host immunity by the pathogen, or cross-reactivity with other bacteria. *B. v. berkhoffii* has co-evolved with domestic dogs and wild canids, and we have previously reported that only 50% of dogs infected with *B. v. berkhoffii* were seroreactive by IFA testing [32]. Therefore, limited antibody response is expected in dogs infected or exposed to this pathogen. Clinical manifestations in immunocompetent dogs and humans infected with this pathogen have been reported, including fatigue, headache, arthritis, muscle pain, neurologic or neurocognitive abnormalities, endocarditis, or epithelioid hemangioendothelioma in humans [24], [33]–[36], and cavitary effusions (pleural effusion, ascitis), endocarditis, cardiac arrhythmias, polyarthritis, anterior uveitis, meningoencephalitis and hemangiopericytoma in dogs [1], [34], [37], [38]. The fact that only asymptomatic dogs infected with *B. v. berkhoffii* were detected in this study is a consequence of the inclusion criteria used, and does not rule out the possibility that sick dogs or humans in Peru may be infected with this pathogen.

The genotype of *B. v. berkhoffii* documented in the six Peruvian dogs in this study was closely related to genotype III originally detected from an endocarditis case in a human in the United Kingdom and in a dog in the USA [24], [25], but differed from the genotype II, which is the most frequent of the four genotypes to be found in dogs and humans in North America [1]. Unfortunately, IFA testing for other genotypes was not available. Lack of concordance between serology and PCR results for *Bartonella* species, as detected in this study, has been previously described in humans and animals, and it is suggested to be associated with an anergic immune response of the host based on the “stealth” properties of *Bartonella* species, or substantial antigenic variation among various *Bartonella* strains, resulting in false-negative IFA assay results [2], [39]. In our study, 39.8% of dogs tested by IFA were seroreactive to both *B. v. berkhoffii* and *B. rochalimae*. Similar results were recently detected from gray foxes in Texas, where 54.5% of 132 foxes were seroreactive to antigens of *B. v. berkhoffii* and *B. clarridgeiae*, which is a suitable antigen marker for *B. rochalimae* detection [40]. Cross reactivity between *B. v. berkhoffii* and *B. rochalimae* cannot be excluded; however, in an experimental infection with these *Bartonella* species in two dogs, no cross reactivity occurred between host anti-*B. v. berkhoffii* or anti-*B. rochalimae* antibodies and antigens from other *Bartonella* species by IFA (titers ≤1∶16) [10]. But when the dog previously exposed to *B. v. berkhoffii* was later experimentally inoculated with *B. rochalimae*, cross reactivity to other *Bartonella* species was documented [10]. These and other limited results suggest that infection or re-infection with multiple species of *Bartonella* could substantially increase the serological cross reactivity between species [10], , which may have occurred within the Peruvian dogs enrolled in this study. However, natural exposure to both pathogens cannot be excluded, as co-infection with more than one species of *Bartonella* species has been documented in several animal species, including dogs and humans [1], [15].

Infections with *B. rochalimae* or *B. v. berkhoffii* have never been described in humans or animals from Peru, even though other known and unknown species of *Bartonella* have been described from small mammals, bats, body lice, fleas and ticks from Peru [22], [42]–[45]. In our study, we documented 10.3% of dogs infected with *B. rochalimae* and 7.7% of dogs infected with *B. v. berkhoffii* from Caraz and Huaraz. In that same region, 127 human cases of bartonellosis were documented in 2008 based on serology methods, with the isolation of *B. bacilliformis* from 11 human specimens [46]. Since humans in Peru are not tested for antibodies against *B. rochalimae* or *B. v. berkhoffii*, their exposure status to these pathogens is unknown. In addition, culture methods used for isolation of *Bartonella* in Peru may bias the detection of *B. bacilliformis* since culture media and temperature used for isolation of *B. bacilliformis* (28°C) are different from *B. rochalimae* and *B. v. berkhoffii* isolation (35°C). Growth differences between isolates of the *Bartonella* genus have been shown *in vitro*, where culture medium and temperature differences as low as only 2°C can select the growth of a given species [47]. Even though *B. bacilliformis* has been a well-established human pathogen in Peru for over a century, our data suggest that other *Bartonella* species may also cause illness in humans in Peru.

The fact that the DNA sequences of *B. rochalimae* and *B. v. berkhoffii* obtained from Peruvian dogs matched 100% and 99.6%, respectively, with *Bartonella* sequences obtained from human fleas (*Pulex irritans*) previously collected from schoolchildren and adults in Peru [22] suggests that human fleas could be the vector for both *Bartonella* species for dogs and humans in that country. The human flea is an aggressive and promiscuous ectoparasite found on a wide range of hosts, including domestic dogs [48], [49]. In addition, *B. rochalimae* was also recently detected in a brown dog tick (*Rhipicephalus sanguineus*) from a dog in the province of Callao [45], located only 25 km (15.5 miles) northwest of San Juan de Miraflores, the site where the largest number of dogs infected with *B. rochalimae* was detected in our study. Both Callao and San Juan de Miraflores are suburbs of the country's capital (Lima), where human and canine population densities and social and economic conditions may favor the multiplication and dissemination of vectors, increasing the risk of human infection. The role of ticks as vector for *B. rochalimae* or *B. v. berkhoffii* in dogs or humans is still unclear. However, clinical studies have associated canine infection with *B. v. berkhoffii* and the presence of *R. sanguineus* ticks [50]. *R. sanguineus* is the most frequent urban tick in tropical regions, and its ability to feed on humans have been documented, including its role in a recent outbreak of Rocky Mountain Spotted Fever in humans in the United States [51].

Cat bites and scratches can transmit *B. henselae* or *B. clarridgeiae* to humans [3]. However, it is still unclear if dogs are capable to transmit *Bartonella* species to humans using similar ways. *Bartonella* DNA was detected in dog saliva [52], and limited case reports have suggested possible direct transmission to humans [53]. In addition, professionals with frequent animal and arthropod exposure, such as veterinarians, veterinary assistants, cattle ranchers and biologists appear to have an occupational risk for *Bartonella* infection [1]. Therefore, the populations in contact with bacteremic dogs should be instructed to prevent arthropod bites, arthropod feces, dog bites, and scratches and direct contact with bodily fluids from infected animals.

In conclusion, our results expand the current knowledge about *B. rochalimae* and *B. v. berkhoffii*, suggesting that asymptomatic domestic dogs in Peru are exposed to these zoonotic pathogens and may be their natural reservoir. Consequently, our results indirectly suggest that the local human population may be exposed to infections with *B. rochalimae* or *B. v. berkhoffii* and support public health actions for vector control in dogs and humans.

## Supporting Information

Text S1 **Alternative Language Abstract.** Translation of the abstract of “Infection of domestic dogs in Peru by zoonotic *Bartonella* species: a cross-sectional prevalence study of 219 asymptomatic dogs” into Spanish by author Cesar M. Gavidia: “La infección de los perros domésticos en Perú por especies zoonóticas de *Bartonella*: Un estudio de prevalencia en 219 perros asintomáticos”.
(DOC)
