# Supplementary material for: Infection of Domestic Dogs in Peru by Zoonotic Bartonella Species: A Cross-Sectional Prevalence Study of 219 Asymptomatic Dogs
Source: PLoS Negl Trop Dis. 2013 Sep 5;7(9):e2393. doi: 10.1371/journal.pntd.0002393 (PMC3764237; doi:10.1371/journal.pntd.0002393)
Supplement: Text S1 — Alternative Language Abstract. Translation of the abstract of “Infection of domestic dogs in Peru by zoonotic Bartonella species: a cross-sectional prevalence study of 219 asymptomatic dogs” into Spanish by author Cesar M. Gavidia: “La infección de los perros domésticos en Perú por especies zoonóticas de Bartonella: Un estudio de prevalencia en 219 perros asintomáticos”. (DOC) [file pntd.0002393.s001.doc]

*Bartonella* spp. son organismos infecciosos transmitidos por artrópodos capaces de causar infección de larga duración en huéspedes mamíferos. Existen 15 especies descritas hasta la fecha que son conocidas por infectar a humanos y ocho de ellas capaces de infectar también a los perros. El objetivo de este estudio fue determinar la prevalencia serológica o molecular de especies de *Bartonella* en perros asintomáticos en Perú con el fin de evaluar indirectamente la posibilidad de exposición humana a las especies zoonóticas de *Bartonella*. Se tomaron muestras de 219 perros sanos que provenían de cinco ciudades y tres pueblos de Perú. Las muestras de sangre con EDTA se obtuvieron de 205 perros y las muestras de suero se obtuvieron de 108 perros. Las muestras de sangre con EDTA fueron usadas para realizar PCR y los productos amplificados fueron secuenciados para la identificación de especies especificas. Los anticuerpos contra *B. vinsonii berkhoffii* y *B. rochalimae* se detectaron por IFA (punto de corte de 1:64). El ADN de *Bartonella* se detectó en 21 de los 205 perros (10%); quince perros fueron positivos a *B. rochalimae*, mientras que seis perros fueron positivos a *B. v. berkhoffii* genotipo III. Se detectó seropositividad para *B. rochalimae* en 67 perros (62%), y para *B. v. berkhoffii* en 43 (40%) de los 108 perros. Se detectaron títulos ≥ 1:256 para *B. rochalimae* en el 19% de los perros y de *B. v. berkhoffii* en el 6,5% de los perros. Este estudio identifica una población de perros expuestos o infectados con especies de *Bartonella* zoonóticas, lo que sugiere que los perros domésticos pueden ser el reservorio natural de estos organismos. Dado que los perros son centinelas epidemiológicos, los seres humanos en las zonas estudiadas pueden estar expuestos a infecciones con *B. rochalimae* o *B. v. berkhoffii*.
